# Research in veterinary sciences in Kazakhstan (2018–2023): developments, gaps and opportunities

**DOI:** 10.3389/fvets.2025.1523732

**Published:** 2025-03-06

**Authors:** Gulzhan N. Yessembekova, Akhylbek K. Kurishbayev, Aruzhan S. Abdrakhmanova, Kuantar D. Alikhanov, Asem Dj. Abenova, Andres M. Perez, Sarsenbay K. Abdrakhmanov

**Affiliations:** ^1^Faculty of Veterinary and Animal Husbandry Technology, S. Seifullin Kazakh Agro Technical Research University, Astana, Kazakhstan; ^2^Kazakh National Agrarian Research University, Almaty, Kazakhstan; ^3^The National Academy of Sciences, Almaty, Kazakhstan; ^4^Faculty of Economics and Business, KU Leuven University, Leuven, Belgium; ^5^Center for Animal Health and Food Safety, College of Veterinary Medicine, University of Minnesota, St. Paul Campus, St. Paul, MN, United States

**Keywords:** Kazakhstan, veterinary sciences, scientific projects, publications, journals, researchers, PhD

## Abstract

Ensuring the welfare of livestock farms, safety of livestock products, control of epizootic situation in Kazakhstan depend on the development of scientific and technical progress. In response to this situation, in order to support the achievement of the strategic development goal, an order for the development of higher education and science in the Republic of Kazakhstan has been implemented with the aim of gradually increasing investment in scientific research to 1% of the country’s gross domestic product (GDP). Our assessment of the scientific output of veterinary sciences in Kazakhstan over the five-year period 2018–2023 demonstrated progress. The total investment in veterinary science has increased to 14 billion KZT, i.e., from 2018, the funding of projects has become annual, previously it was once every 3 years. The consequence of this transformation was a 5-fold increase in the number of published articles for example, in 2022–2023 their number reached 50 compared to 2018–2019, where the number was barely 10. Despite the positive trend in veterinary science in recent years there are still gaps in the form of inadequate funding (only 20 funded projects per year with an average allocation of <200,000 USD per project), the productivity of the scientific community has been lower than expected: 91 peer-reviewed publications were published in first quartile journals over 5 years, which is an average of one publication in first quartile journals per year for every 32 PhD researchers (with a total of 584 PhDs), concentration of science only in large cities of Kazakhstan (Astana, Almaty), scientific developments of scientists are not commercialized and have no feedback with production. Thus, there is a need to continue to improve the effectiveness of veterinary research in combination with education and retraining, as well as increasing the participation of underserved regions and communities in the country.

## 1 Introduction

Kazakhstan, a land-locked territory in Central Asia, is the ninth-largest country in the world based on its geographical extent. Historically inhabited by nomadic communities, the country experienced unprecedented political and economic changes during Soviet times, which were followed by a new period of social transformation subsequent to the dissolution of the Soviet Union. Compared to other countries in the region, Kazakhstan performs relatively well in terms of social and economic development. Kazakhstan ranks among the top third of countries worldwide in terms of its Human Development Index (HDI), which is a combined indicator aimed at characterizing human development and that considers factors such as life expectancy, literacy level of the population, and standard of living, assessed through gross national income per capita at purchasing power parity (PPP) in US dollars (1). Among member countries of the Eurasian Economic Union (EAEU) and the Commonwealth of Independent States (CIS), Kazakhstan ranks second, in terms of its HDI, only after the Russian Federation (2). In line with the Sustainable Development Goals adopted by the United Nations in 2015, the strategic goal of the Republic of Kazakhstan is to achieve high-quality and sustainable economic growth by 2025. The expectation of such strategic goal is to improve the well-being of people based on increased competitiveness of business and human capital, technological modernization, improvement of the institutional environment and minimal negative impact on nature (3).

Comparatively, however, Kazakhstan is not performing so well in terms of its Global Innovation Index (GII), which ranks world economies according to their innovation performance. Consisting of approximately 80 indicators grouped by innovation inputs and outputs, the index takes into account various aspects of innovation. The GII represents the ratio of costs and benefits, which allows for the objective assessment of the effectiveness of efforts to develop innovation in a particular country. The GII is considered one of the most important indexes for assessing the level of scientific, technical, and innovative development of countries around the world (4–6). In 2022, Kazakhstan’s GII ranked 83rd among 132 countries and 4th in the EAEU, behind the Russian Federation, Belarus, Armenia, and Uzbekistan.

In response to this situation and in order to support the achievement of its strategic goal for development, Kazakhstan implemented an Order for the Development of Higher Education and Science in the Republic of Kazakhstan (2023–2026). The objective of that order is to increase the global competitiveness of Kazakh scientific research and to increase its contribution to solving applied problems at the national level, including the gradual increase of research and development investments from all sources to 1% of the country’s GDP (7).

The objective of this paper was to evaluate the metrics of the research system for veterinary sciences in Kazakhstan over the last 5 years (2018–2023). The results presented here will help evaluate the evolution of veterinary research in Kazakhstan, creating foundational knowledge and metrics for the continuous evaluation of the system, and providing background information to evaluate the impact of the Order for the Development of Higher Education and Science in the Republic of Kazakhstan in veterinary sciences.

## 2 Historical context

In the Soviet period, research in the field of veterinary science in the Republic of Kazakhstan was carried out by researchers affiliated with specialized research institutes, such as the Kazakh Research Institute and the Agricultural Research Institute, as well as five higher education institutions. During this period, because of the nature of the Soviet regime, science and education took place in a closed society. Publication of research results in internationally accessible peer-reviewed scientific journals was rare for a number of reasons, including political unwillingness to engage with the Western world, limited English language knowledge, and difficulty connecting with the international research community. Research results took the form of monographs, theses, and manuscripts that, in some cases, were published in scientific journals available at the Kazakh and/or Soviet Union levels.

The first years after Kazakhstan gained independence in 1991 were characterized by a severe economic crisis that had a negative impact on veterinary research. Many researchers left the country, there was a large outflow of personnel to other areas of activity, and recent graduates were not interested in pursuing a scientific career due to the uncertain prospects. As an example, a PubMed search of the keyword “Kazakhstan” of articles published in English language targeting non human animals retrieved only 14 scientific articles published in 2005. All those articles were reviews or descriptive articles of the presence of pathogens or diseases, written in collaboration with scientists associated with institutions outside the country, in many cases, where Kazakh researchers have migrated, suggesting that the research generated in Kazakhstan institutions was very limited.

In response to that crisis, the scientific system was restructured, including measures such as the creation of a competitive grant process to which any institution in the country could apply, the Bolashak (“future”) program that provides funding for training of scientists at international research centers, and grant funding competitions specific for young (<40 year old) scientists. In the following sections we discuss the changes in scientific production observed over the last 5 years.

## 3 Scientific funding and production

Funding for research projects in Kazakhstan are mostly provided by the National Center for Science and Technology (NCST). The duration of projects is typically 3 years, and requests for proposals are issued annually. Since 2018, there have been five funding cycles. The average number of NCST-funded projects in the areas of veterinary science and food safety and nutrition per funding cycle were 20 (minimum 9, maximum 31) and 14 (minimum 5, maximum 22), respectively. The average funding allocated to each project was 82,817,180 tenge (~USD 173,000) for a total investment of >14 billion tenge (USD >29 million) over the 5-year period.

Areas funded in the field of veterinary sciences included the development of vaccines and biologicals (26% of the projects), of diagnostic tests and products (26% of the projects), epidemiological or pathogenic evaluation of infectious agents (26% of the projects), and molecular and genetic studies (22% of the projects). In the area of food safety and nutrition, 54% of the funded projects were aimed at developing food additives and improving human nutrition, 19% were focused on the development of animal feed additives, 15% on the study of the properties of food, and 12% of the projects focused on veterinary and sanitary food control and safety.

Scientific production emerging from those projects included the publication of 249 manuscripts in peer-reviewed journals between 2018 and 2023. Only 36.5% of those papers were published in journals on the top 25% (first quartile) cite score (referred to as quartile one, or Q1). The annual number of published papers increased from 4 in 2018 and 6 in 2019 to 28 in 2022 and 22 in 2023 (8). Five organizations concentrated 70% (88/126) of the nationally funded projects and 49% (156/319) of the patents registered in the areas of veterinary science and food safety and nutrition in Kazakhstan (Table 1).

**Table 1:** Scientific production of the five most active research organizations in the fields of veterinary science and food safety and nutrition in Kazakhstan (2018–2023).

Organization	Number of projects		Patents	Number of faculty with a PhD degree (9–17)	Scientific papers	
	Veterinary science	Food safety			Total	In the first quartile (Q1) site score
S. Seifullin Kazakh Agro Technical Research University	13	12	14	79	48	25
National Center of Biotechnology	16	4	5	87	11	11
Research institute for biological safety problems	17	0	20	152	14	12
Research and Production Center of Microbiology and Virology	12	3	10	40	19	4
Kazakh National Agrarian Research University	6	7	107	95	61	29
Total top five organizations (and percentage of total in Kazakhstan)	88 (69.8%)		156 (48.9%)	453 (77.6%)	153 (61.4%)	81 (89.0%)
Total in Kazakhstan	126		319	584	249	91

Most (89.7%) of the 212 articles retrieved from the Scopus database focused on infectious diseases, with only 22 (10.3%) manuscripts dedicated to the study of non-infectious diseases. This asymmetry may be explained, at least in part, to the legacy of Soviet times, in which research institutes in the Republic of Kazakhstan were mainly aimed at combating infectious diseases of animals, and certainly represents a gap that should be covered in the country.

## 4 Gaps and perspectives

This manuscript summarizes metrics of veterinary sciences research in Kazakhstan, as part of the country’s national policy intended to enhance the performance of scientific research in the country and, ultimately, support its development. Over the assessed period (2018–2023), there seems to be a positive evolution in scientific production, as suggested by the 5-fold increase in the number of papers published in 2022–2023 (*n* = 50) compared to 2018–2019 (*n* = 10). Such positive impact has been predominantly led by five research and academic organizations that received 70% of the funded projects and were responsible for 89% of the papers published in Q1 peer-reviewed journals in the country (Table 1).

The country’s policy intended to enhance scientific productivity was implemented by creating a competitive process for allocating funding for research projects, which goes beyond the allocation of discretionary funding for operation and maintenance. The competitive process, implemented through the National Center for Science and Technology, represents a substantial change compared to practices for the allocation of funding followed in Soviet times. This initiative is commendable, as it opens the opportunity for competition among researchers and institutions, which is a fundamental feature of modern scientific research. However, the amount of funding allocated to veterinary research in Kazakhstan still seems to be lower than what would be required to support the ultimate goal of development, considering the size of Kazakhstan economy (20 projects funded per year at an average allocation of <USD 200,000 per project).

The productivity per funded project, and funding allocated for research, seems to still be lower than the desired in the country. For example, there have been 91 peer-reviewed publications in Q1 journals emerging from 126-funded project—i.e., on average, less than one publication emerging per funded project (8). Similarly, the productivity of the scientific community seems to be lower than expected, with 91 peer-reviewed publications published in Q1 journals over 5 years for a critical mass of 584 researchers with a PhD degree in the country—leading to an average of one publication in Q1 journals per year every 32 researchers with a PhD degree.

These figures illustrate, at the same time, the need and opportunity to continue to make progress in developing the structure of the scientific system for veterinary research in Kazakhstan. The process for allocation of research funding, for example, should be accompanied by actions intended to increase the competitiveness of academic institutions other than the five top leading organizations. Those efforts should aim at supporting the research capabilities and competitiveness of institutions located outside Almaty and Astana, the two most populous cities of the country. Kazakhstan is a vast country, with various distinct regions in terms of demographics, ecology, and geography, leading to diverse requirements. There is a need to enhance the participation of institutions serving those locations to address local needs and provide support to the development of communities throughout the country.

In line with the requirement of enhancing the geographical scope of veterinary research in Kazakhstan, there is also a need to continue supporting the preparation and training of Kazakh scientists. Specifically, there is a need for increasing the capacity of researchers in the proficient use of the English language applied to scientific writing and their understanding of the dynamics of the peer-reviewed publication process. Much of the scientific production in Soviet times took the form of monographs, reports, and dissertations. Publication of research results in peer-reviewed journals was uncommon and, for that reason, the process, its implications, and features, is still insufficiently understood by the scientific community of the country. Subsequently, there is a need to promote the education of the new generation of veterinary researchers in the science of peer-reviewed publication, as a prerequisite to enhance the participation of the country in the global share of scientific publishing.

Additionally, there are disciplinary limitations in the country. Most notably, certain disciplines within veterinary sciences (such as applied epidemiology, statistics, and molecular biology) were not sufficiently taught during Soviet times in the country. For that reason, those disciplines still seem to be on their infancy in Kazakhstan. Because of the central role that those disciplines play in hypothesis-driven research at the population level in veterinary sciences, such condition results in a limitation of the scientific productivity of researchers in the country.

There is also a need to give direction to the subjects or themes funded for research in Kazakhstan. To a greater extent, funded projects are typically basic in nature, with most (>50%) of the funded projects intended to develop new vaccines or diagnostic tools, or supporting foundational molecular research. Those projects are primarily intended to developing groundbreaking research, which typically are high risk in terms of the probability of success. In turn, there is a weak connection between scientific research, the field needs of veterinary practice, and it is served communities. Much of the resulting research is theoretical or basic in nature, with limited field impact. There is a need for prioritizing the funding of research projects and initiatives with a direct impact in the field and throughout the country, with the objective of enhancing the quality of veterinary practices and the social development of all the country’s regions.

Three most urgent recommendations intended to continue supporting the development of veterinary research in Kazakhstan could include:

Supporting centers of excellence for research in remote locations and under-served communities to promote the development of science outside the main cities of the country.
Favoring the effective recruitment and retention of promising and qualified researchers and research personnel, as well as their training on transversal aspects of research, such as study design, epidemiological and statistical analysis, proficiency in the use of the English language, and scientific writing.
Enhancing the availability of funding to support research at universities and launch funding opportunities to support the creation of research infrastructure, increasing the funding available during the first year of research to facilitate the acquisition of the required equipment.

## 5 Conclusion

In summary, the assessment of the scientific performance of veterinary sciences in Kazakhstan during the 2018–2023 five-year period demonstrates the country’s progress in its efforts to modernize the design, funding, and productivity of research programs in the country. The objective of the Order for the Development of Higher Education and Science in the Republic of Kazakhstan (2023–2026) was to increase the global competitiveness of the country’s science with the ultimate objective of contributing to solving applied problems in Kazakhstan.

Although important progress has been made, there is still a need and opportunity to continue deepening the impact of veterinary research through a combination of education and training and increasing the participation of underserved regions and communities in the country.

## Data Availability

The raw data supporting the conclusions of this article will be made available by the authors, without undue reservation.
